# Large regional variation in endovascular thrombectomy rates for acute ischemic stroke in Sweden

**DOI:** 10.1177/23969873251347098

**Published:** 2025-06-16

**Authors:** Johan Wassélius, Emma Hall, Alex Szolics, Fabian Arnberg, Hozan Radhi, Mia von Euler, Per Wester, Teresa Ullberg, Tobias Cronberg, Nicklas Ennab Vogel, Magnus Esbjörnsson, Fredrik Jonsson, Tommy Andersson, Bo Norrving, Björn M Hansen

**Affiliations:** 1 Department of Radiology, Skåne University Hospital, Lund, Sweden; 2 Stroke Imaging Research Group, Department of Clinical Sciences Lund, Lund University, Lund, Sweden; 3 Department of Neurology and Rehabilitation,School of Medicine, Örebro University, Örebro, Sweden; 4 Department of Neuroradiology, Karolinska University Hospital, Solna, Sweden; 5 Department of Clinical Neuroscience, Karolinska Institutet, Stockholm, Sweden; 6 Department of Public Health and Clinical Medicine, Umeå University, Umeå, Sweden; 7 Department of Clinical Science, Karolinska Institutet Danderyds Hospital, Stockholm, Sweden; 8 Department of Neurology, Skåne University Hospital, Lund, Sweden; 9 Neurology, Department of Clinical Sciences Lund, Lund University, Lund, Sweden; 10 Department of Medical and Health Sciences, Linköping University, Linköping, Sweden; 11 Department of Internal Medicine, Hässleholm Hospital, Hässleholm, Sweden

**Keywords:** Acute ischemic stroke, endovascular thrombectomy, effectiveness, implementation, key performance indicators (KPIs)

## Abstract

**Introduction:**

Endovascular thrombectomy (EVT) is a significant improvement in the care of acute ischemic stroke (AIS) patients, but only a small portion of patients receive treatment. Our aim was to analyze EVT implementation in Sweden according to a set of key performance indicators (KPIs) for *procedural* and *implementational effectiveness*.

**Methods:**

A nationwide prospective registry-based observational study using data from 2018, 2020, and 2022 from the Swedish quality registries for stroke care (Riksstroke and EVAS) and official population statistics. Effectiveness was analyzed using a set of predefined KPIs. To describe procedural and implementation effectiveness in a single comprehensible measure population success rate was derived by multiplying the EVT rate with successful recanalization.

**Results:**

Between 2018 and 2022 EVTs in Sweden increased from 874 to 1474 procedures per year. Correspondingly, the EVT rate (EVT/AIS) increased from 4.1% to 7.3%. Implementation was heterogenous with a six-fold difference between the highest and lowest regions. EVT rates were generally highest in regions with comprehensive stroke centers (CSCs). Procedural effectiveness were similar between all CSCs. The population success-rate increased from 3.4% to 6.4% during the period with large differences between CSCs (range 3.4%–12.4%, in 2022).

**Conclusions:**

By including KPIs for procedural and implementational effectiveness, it is possible to evaluate EVT implementation for the entire stroke population, which is the ultimate objective for healthcare. The population success-rate is capturing procedural implementation effectiveness in a single measure comprehensible for all stake holders and facilitate comparisons over time and between regions, even between regions with different stroke incidence.

## Graphical abstract

**Graphical abstract:**
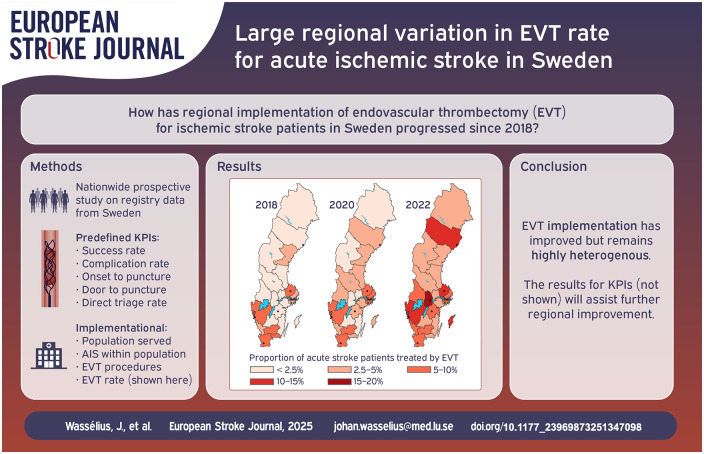

## Introduction

Endovascular thrombectomy (EVT) has marked a significant shift in the management of acute ischemic stroke, substantially improved functional outcome and has rapidly been introduced as standard of care. Following the initial successful randomized controlled trials (RCTs) for large vessel occlusion (LVO) in the anterior circulation,^1,2^ treatment has been expanded to the later time window,^3^ the posterior circulation,^4^ and lately to patients with more extensive ischemic damage at presentation.^5^ Additional studies have shown that the benefit of EVT extends to the very old patients^6^ and to patients with extensive comorbidity burden.^7^ Nonetheless, the portion of stroke patients that receive any recanalizing treatment at all remains as low as 5% in a rather recent global estimation.^8,9^

In medicine, *efficacy* typically describe how well an intervention produces the expected result under ideal circumstances, such as in well conducted randomized clinical trials.^1,3–5^ Effectiveness on the other hand measure the benefit of the intervention under real-world conditions in routine healthcare.^10^ In this work we define effectiveness of the EVT procedure as *procedural effectiveness*, whereas how well the treatment is implemented into the community is defined as *implementational effectiveness*.

Procedural effectiveness would thus capture the ability to provide timely treatment with a high success-rate and low complication-rate.

Implementational effectiveness would include identification of a potential EVT cases based on stroke symptom, triaging to the correct level of care and offering EVT treatment to all eligible candidates following guidelines when applicable, scientific evidence and clinical judgment where guidelines are not applicable.

Although both are desirable, implementational effectiveness is more important to maximize the benefits of EVT in the entire stroke population. This aspect of EVT implementation was highlighted early in the EVT era as the “denominator fallacy,” by which overly strict selection criteria may seemingly produce excellent results when looking only at the treated population but may not drive healthcare to produce the best possible care to the stroke population as a whole. This aspect is of utmost importance in clinical practice^11^ where entire regional or national populations are served.

National quality registry data are instrumental to monitor the effectiveness of EVT, identifying bottlenecks and factors associated with procedural success or failure in routine healthcare, as well as benchmark performance between comparable institutions.

Our aim with the present study was to analyze the procedural and implementational effectiveness of EVT over time on a national and regional level in Sweden according to a predefined set of key performance indicators (KPIs).

## Methods and material

### Study design

A nationwide prospective registry-based observational study was conducted based on data from the Swedish quality registry for stroke care (Riksstroke), the Swedish EndoVAscular treatment of Acute Stroke (EVAS) registry and Statistics Sweden. This study was approved by the Swedish Ethical Review Authority (#2019-00678) and informed consent was waived by the Authority.

### Data sources

Regional population data were collected from Statistics Sweden, the governmental provider of official statistics, including national and regional demographics.

Data on stroke incidence and EVT treatments specified for each region were collected from Riksstroke. Riksstroke collects data from all 72 hospitals in Sweden managing acute stroke care with a consistent coverage of >90% for all strokes.

Data on EVT characteristics such as occlusion location, procedural and treatment details, as well as procedure-related complications were obtained from EVAS – the Swedish quality registry for EVT procedures with a coverage >95%. The caseload for the interventionalists performing EVTs in 2018, 2020, and 2022 was also attained from EVAS.

### Participants

All patients treated endovascularly by thrombectomy in Sweden during 2018, 2020, and 2022 were included in this study.

Sweden is divided in 21 independent regions that are the main healthcare providers. All acute stroke care is provided by hospitals organized within these regions. During 2018 and 2020 there were six comprehensive stroke centers (CSC) in operation. In 2022, an additional CSC was established, increasing the total to seven. CSCs generally serve all primary stroke centers (PSC) in their own healthcare region as well as any number of PSCs in surrounding regions without a CSC of their own. For analysis of the larger collaborations between a CSC and its surrounding regions, the referring regions were grouped with the region of the CSC to which they referred most of their patients that particular year.

Patients were included regardless if they were directly triaged to the CSC, or if they were transferred from a PSC, but the data was used to calculate the portion of patients that were directly triaged to CSC.

### Definitions of effectiveness

In medicine, we typically distinguish between the *efficacy* and the *effectiveness* of an intervention. Efficacy describe how well the intervention produces the expected result under ideal circumstances, most often in well conducted randomized clinical trials.

Effectiveness on the other hand measure the benefit of the intervention under real-world conditions in routine healthcare.^11^ We define effectiveness of the EVT *procedure* in routine healthcare as *procedural effectiveness*, whereas how well the treatment is implemented and provided to the community is defined as *implementational effectiveness*.

### Variables and definitions

EVT was defined as any initiated EVT procedure.

Technical success was defined as a modified treatment in cerebral infarction (mTICI) score of 2B–3 on a digital subtraction angiography (DSA) after EVT.^12^

Procedural complications included any reported complications related to the EVT procedure.

The included Key Performance Index for EVT procedural effectiveness was *procedural success rate* (measured as the frequency of procedures resulting in a mTICI score of 2B-3), *complication rate* as a measure of safety (frequency of any reported procedural complication), stroke *onset to arterial* puncture (hours and minutes) as an overall measure of the stroke pathway including geographical aspects, *door to arterial puncture* (minutes) as a measure of the team coordination, and the *direct triage rate* (the portion of EVT cases treated at the CSC who had been triaged directly to that hospital) as an effectiveness measure of the prehospital triage system.

Key Performance Indicators (KPIs) for implementational effectiveness were prespecified and included the yearly size of the population served by each CSC, the yearly number of AIS within the served population, and the yearly number of EVT procedures within the served population. We chose to relate the number of EVT procedures to the number of AIS cases in the served population (*EVT rate*), in line with the ESO stroke action plan KPI 7b,^9^ instead of the population size, to compensate for regional differences in yearly stroke incidence.

To combine procedural and implementational effectiveness into one single effectiveness KPI, we defined the novel KPI “population success-rate” as the EVT-rate (EVT/AIS) multiplied by the procedural success-rate (mTICI ⩽2B).

#### Statistical analysis

Data are presented as means (normally distributed data) or medians (not normally distributed data) with interquartile ranges (IQR) or as simple proportions. Statistical analysis was done using GraphPad Prism version 10.2.3.

## Results

**Figure 1.**
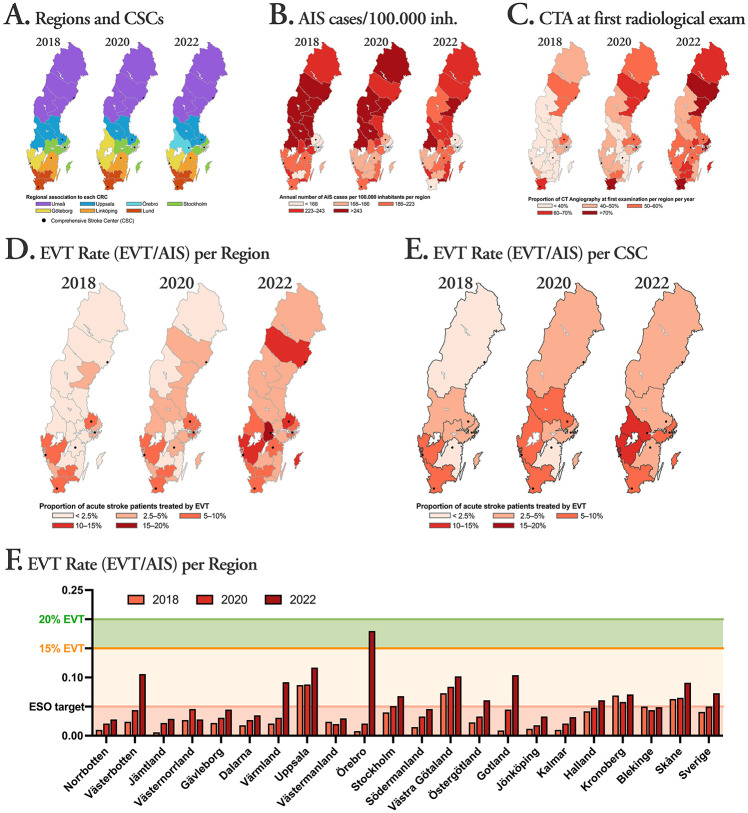
Panel (A) Color-coded catchment areas comprised of 21 regions in total for the CSCs indicated by black dots (six in 2018 and 2020, seven in 2022). Panel (B) AIS cases per 100,000 inhabitants per region. Panel (C) Frequency of CTA at the initial radiological examination per region. Panel (D) EVT rate (measured as EVT/AIS) per region. Panel (E) EVT rate per CSC including all regions referring patients to that CSC. Panel (F) Bar chart showing the EVT rates for each region (from north to south).

The Swedish population is largely concentrated to three major urban regions as illustrated in Supplemental Figure 1 where each region is labeled based on its portion of the total population. During the time-period studied, the regional referral pattern changed somewhat mainly due to the start of one new CSC (Örebro), as shown in Panel (A), Figure 1. The total number of registered interventionalists performing EVT increased during the study period, from 22 in 2018 to 25 in 2020 and finally to 31 in 2022. Of these operators the majority performed more than 10 EVTs (2018: *n* = 20; 2020: *n* = 22; 2022: *n* = 27) while a minority performed more than 50 EVTs (2018: *n* = 6; 2020: *n* = 6; 2022: *n* = 12).

The annual number of AIS stroke cases varies considerably between healthcare regions, as shown in Panel (B), Figure 1, likely due to underlying factors such as the age distributions.

Primary radiological investigation was done by CT in a vast majority of AIS patients in Sweden (>99%). The portion of patients that had CT-angiography (CTA) done at the initial radiological examination is shown in Panel (C), Figure 1.

The EVT rate within the population served is shown for each CSC and year in Panel (D), Figure 1, and for each CSC-region and year in Panel (E), Figure 1.

In Table 1 we have listed the predefined KPIs associated with procedural effectiveness; procedural success rate; complication rate; median onset to groin time; door to groin time; and the direct triage rate. Procedural success-rate per CSC per year is ranging from 78% to 94% with an increase nationwide from 83% to 88% from 2018 to 2022. Complication rate per CSC per year is ranging from 1% to 9% and is relatively unchanged nationally at 5%. The median onset-to-groin-times (hh:mm) range from 2:34 to 4:51 per CSC per year and are relatively unchanged over time, with the exception of Gothenburg that have managed to decrease the median onset-to-groin-time with more than 30 min, from 3 h and 7 min in 2018 to 2 h and 34 min in 2022. The door-to-needle-times per CSC per year ranged from 9 to 30 min and are relatively unchanged over time. The direct triage rate per CSC per year ranged between 12% and 54% and has increased over time at several centers (Umeå, Stockholm, and Linköping) as well as nationally.

**Table 1.** Pre-selected procedural effectiveness KPIs for each CSC for 2018, 2020, and 2022; procedural success rate (defined as mTICI ⩾2B), procedural complication rate, median onset to puncture time (hh:mm), median door to puncture time (hh:mm), and the direct triage rate.

CSC	Success rate			Complication rate			Onset to puncture time (median)			Door to puncture time (median)			Direct triage rate		
	2018	2020	2022	2018	2020	2022	2018	2020	2022	2018	2020	2022	2018	2020	2022
Umeå	78%	83%	83%	9%	2%	1%	4:38	4:51	4:47	00:25	00:25	00:22	40%	19%	33%
Uppsala	87%	83%	91%	3%	3%	5%	3:42	4:10	3:09	00:24	00:22	00:20	49%	54%	51%
Örebro			94%			3%			3:32			00:22			35%
Stockholm	82%	88%	87%	5%	3%	3%	2:36	2:52	2:43	00:20	00:23	00:21	52%	47%	42%
Göteborg	82%	94%	94%	6%	5%	5%	3:07	2:45	2:34	00:09	00:13	00:11	53%	51%	52%
Linköping	91%	87%	81%	4%	2%	2%	4:07	4:19	4:30	00:30	00:28	00:26	50%	38%	18%
Lund	82%	81%	81%	6%	8%	7%	3:22	3:30	3:29	00:18	00:22	00:20	20%	19%	12%
Sweden	83%	87%	88%	5%	5%	4%							43%	40%	36%

In Table 2 we have listed the predefined KPIs associated with implementational effectiveness, that is, how well EVT is implemented in relation to the population served; the size of the population served by each CSC for each year; the AIS incidence in that population for each year; the number of EVT procedures within that population and related to the AIS incidence within that population.

**Table 2.** Pre-selected implementational effectiveness KPIs for each CSC for 2018, 2020, and 2022; the population served by each CSC, the AIS frequency within the population served, the number of EVT procedures in each CSC, the EVT rate and the population success rate (calculated as (EVT/AIS) × success rate).

CSC	Population served			AIS within population served			EVT procedures			EVT rate			Population success-rate		
	2018	2020	2022	2018	2020	2022	2018	2020	2022	2018	2020	2022	2018	2020	2022
Umeå	896,384	898,515	901,407	2358	2277	2168	40	81	109	1.7%	3.6%	5.0%	1.3%	2.9%	4.2%
Uppsala	1,231,574	963,572	1,257,039	2870	2147	2653	102	115	131	3.6%	5.4%	4.9%	3.1%	4.5%	4.5%
Örebro			591,748			1424			188			13.2%			12.4%
Stockholm	3,281,802	3,334,299	2,803,766	5923	5520	4591	181	228	324	3.1%	4.1%	7.1%	2.5%	3.6%	6.1%
Göteborg	1,709,814	2,017,328	1,758,656	3553	3899	3109	278	299	342	7.8%	7.7%	11.0%	6.4%	7.2%	10.4%
Linköping	1,067,078	1,078,178	1,088,736	2151	2058	2272	32	47	96	1.5%	2.3%	4.2%	1.3%	2.0%	3.4%
Lund	2,051,086	2,087,403	2,120,204	4325	4152	3902	241	238	284	5.6%	5.7%	7.3%	4.6%	4.7%	5.9%
Sweden	10,237,738	10,379,295	10,521,556	21,180	20,053	20,119	874	1008	1474	4.1%	5.0%	7.3%	3.4%	4.4%	6.4%

Table 2 shows the population served by each CSC per year, the number of AIS cases per year and the number of EVT procedures within its population for each CSC per year. The EVT rate is increasing nationwide from 1.7% in 2018 to 5.0% in 2022. The EVT rate is increasing over time at all CSC, but with substantial differences, for example the highest EVT rate in 2018 was more than 5 times higher than the lowest (Göteborg 7.8% vs Linköping 1.5%), and more than 3 times as high in 2022 (Örebro 13.2% vs Linköping 4.2%).

The *population success-rate* (Table 2; EVT/AIS multiplied by the procedural success-rate), thereby combining procedural and implementational effectiveness into a single comprehensible KPI. Over the time period the population success-rate of Sweden has almost doubled from 3.4% to 6.4%. Similarly to the crude EVT rate, all centers show an increasing population success-rate over the period, but with large differences between centers. In 2018 the CSC with the highest population success-rate was almost 5 times higher than the lowest (Göteborg 6.4% vs Umeå and Linköping 1.3%), and in 2022 more than 3 times higher (Örebro 12.4% vs Linköping 3.4%).

## Discussion

In this study we use national quality registry data to study the implementation of EVT on national level finding that there is considerable larger variation in implementational EVT effectiveness compared to procedural effectiveness, including a three-fold difference in EVT-rate between the highest and lowest CSC, and a six-fold difference in EVT-rate between the highest and lowest individual regions.

Endovascular thrombectomy has rapidly and substantially shifted the management of AIS with substantial improvements in functional outcome for the most severe AIS cases caused by LVOs.^1,3,4^ The treatment effect is one of the largest seen in medicine, which should inspire healthcare organizations and communities at large to maximize its use and to ensure that all eligible patients are offered the treatment.

Even so, systematic reports on EVT implementation are relatively scarce, especially in comparison to the literature on EVT efficacy. In fact, it is not well established how large the potentially treatable portion of AIS really is, especially within the first hours of onset, for the simple reason that a majority of cases are still not identified and examined within the first hours. Systematic evaluations based on large AIS populations examined by CTA within the first hours of onset suggest that at least 15%–20% of AIS patients may be eligible for EVT.^13–16^

Our analysis of the EVT effectiveness in routine care in Sweden shows a gradual improvement in over time.^17^ It also demonstrates how shortcomings in the early years, such as the low use of CTA at the first radiological examination, have been at least partly overcome. In 2022, the majority of patients had a CTA included at the first radiological examination, even though there is room for further improvement.

In 2018, 3 years after the groundbreaking trials that proved the superiority of EVT in addition to IVT, EVT implementation was as low as 4.1% nationwide, mainly concentrated to the three urban areas (Figure 1, panel (E)). Even though almost all regions show improved EVT implementation over time, the regional differences are and remain high, in particular for implementational effectiveness KPIs. The reasons for this are likely multiple and may vary between regions, for example the use or non-use of modern stroke networks/communication platforms and adherence to guidelines and new scientific data are all important factors. Availability of operators or capacity for training new operators and new centers may be another limiting factor,^18,19^ especially in regions where the transportation times to CSC are very long. All these factors aside, we believe that a major contributing factor is that KPIs for implementational effectiveness have not had the same attention as procedural effectiveness. It would be hard to imagine that a three-fold difference in procedural success rate between CSCs would go unnoticed in the same way that the three-fold difference in EVT-rate has done.

It is especially true for implementational effectiveness, since in our daily work we generally don’t see the patients who we fail to identify therefore we don’t experience bad implementation in the same way as we would experience bad EVT procedures.

EVT implementation is often measured as EVT/100,000 inhabitants, which may be reasonable when comparing regions with a similar AIS frequency, but since there are large regional variability in AIS frequency, the EVT rate (EVT/AIS) has been found to be a more relevant measure of effectiveness by the ESO Stroke Action Plan.^9,19^ This is illustrated by a two-fold difference in AIS frequency between the highest and lowest regions in Sweden (as shown in Figure, panel (B)), something that should be reflected in effectiveness metrics.

Since implementational effectiveness measures are generally more important to the population, the argument could be made that the EVT rate is the single most important EVT metric, reflected in the fact that it is the only EVT metrics in the ESO Stroke Action Plan.^9^ However, since procedural effectiveness is also of the greatest importance for the served population, it may make sense to combine implementational and procedural effectiveness into one single KPI, such as *population success-rate*, (EVT/AIS × procedural success-rate), which incentivizes ambitious implementation as well as effective procedures.

Clinical outcome, typically measured by the modified Rankin scale (mRS) at 3 months following the stroke, is obviously the paramount objective of stroke care, but may be less suitable as a metric to evaluate EVT effectiveness in routine care since the clinical outcome is highly affected by the case-selection and there is no control population as in the RCTs. For example, a CSC with a high portion of patients with more extensive ischemic damage at presentation^5^ would seemingly to do worse compared to a CSC with a low portion of that patient group. The metric could therefore risk becoming an incentive for over-selection (cherry-picking), which would not benefit the stroke population as a whole. This is the rationale why we chose to exclude mRS and mortality as KPIs in this study.

By analyzing each CSC together with its entire catchment area over time using the proposed KPIs, we can illustrate the overall strengths and challenges for each CSC and compare it to others. For example, it is clear that the geographically largest area served by one CSC (Umeå), likely would benefit from additional CSCs in its geographical catchment, since the marked improvements within its own region are not matched in the surrounding referring regions, and it is possible to estimate the number of cases at such new centers. It is encouraging to see that a new CSC (Örebro) could deliver excellent EVT service already from the start, and increase EVT implementation in its own region with a 22-fold increase in EVT rate over the period (0.8% in 2018 vs 18% in 2022), accompanied with an increase in its neighboring regions too, providing first-rate EVT service on the population level as shown by the population success-rate KPI.

Since the EVT rates are highly variable between the 21 regions, benchmarking and implementation of national standards, appear to be important to achieve more equal stroke care throughout the entire country. National quality registries will have an important role to monitor EVT implementation and key enabling factors such as the number of EVT operators to ensure that bottlenecks are identified and mended. A national perspective should also be used when evaluation the implementing of potential improvements of the stroke care, such as the use of modern tele-stroke networks or prehospital triage systems, and such regional improvements could thereby rapidly be implemented on national level.

### Strengths and limitations

The major strength of this analysis is that it is based on nationwide data with high coverage during a dynamic time with respect to implementation of EVT.

This study has several limitations. The mTICI used to determine procedural success rate as well as the complication rate, is self-reported by the operator or someone else at the performing center, and over- and underreporting may represent a systematic error. Previous validation studies have shown that under- and overreporting are more common in mTICI 2c and 3, and less common between mTICI 2a and 2b.

The methodological choice to assign all patients from a referring region to one CSC may cause a systematic error in the analysis.

Survival and functional outcome are not included in this analysis, which is a limitation. The main reason for us not to include that as KPIs, even though it is of paramount importance, is that it may reward over-selection (cherry-picking) and therefore we did not find it suitable in this particular analysis.

## Conclusion

EVT for AIS is gradually increasing in Sweden but the implementation is highly heterogenous between regions. The major challenge now is to expand EVT to all eligible patients and to achieve this, implementational effectiveness metrics needs additional focus in CSC monitoring and benchmarking on a national level.

KPIs used to monitor EVT should be comprehensible to all stakeholders and include implementational effectiveness metrics, such as EVT-rate. The population success-rate combines procedural and implementational effectiveness into one single metric.

## Supplemental Material

sj-tif-1-eso-10.1177_23969873251347098 – Supplemental material for Large regional variation in endovascular thrombectomy rates for acute ischemic stroke in Sweden Supplemental material, sj-tif-1-eso-10.1177_23969873251347098 for Large regional variation in endovascular thrombectomy rates for acute ischemic stroke in Sweden by Johan Wassélius, Emma Hall, Alex Szolics, Fabian Arnberg, Hozan Radhi, Mia von Euler, Per Wester, Teresa Ullberg, Tobias Cronberg, Nicklas Ennab Vogel, Magnus Esbjörnsson, Fredrik Jonsson, Tommy Andersson, Bo Norrving and Björn M Hansen in European Stroke Journal
