# Supplementary figures and images for: Large regional variation in endovascular thrombectomy rates for acute ischemic stroke in Sweden
Source: Eur Stroke J. 2025 Jun 16;10(4):1320–7. doi: 10.1177/23969873251347098 (PMC12174580; doi:10.1177/23969873251347098)

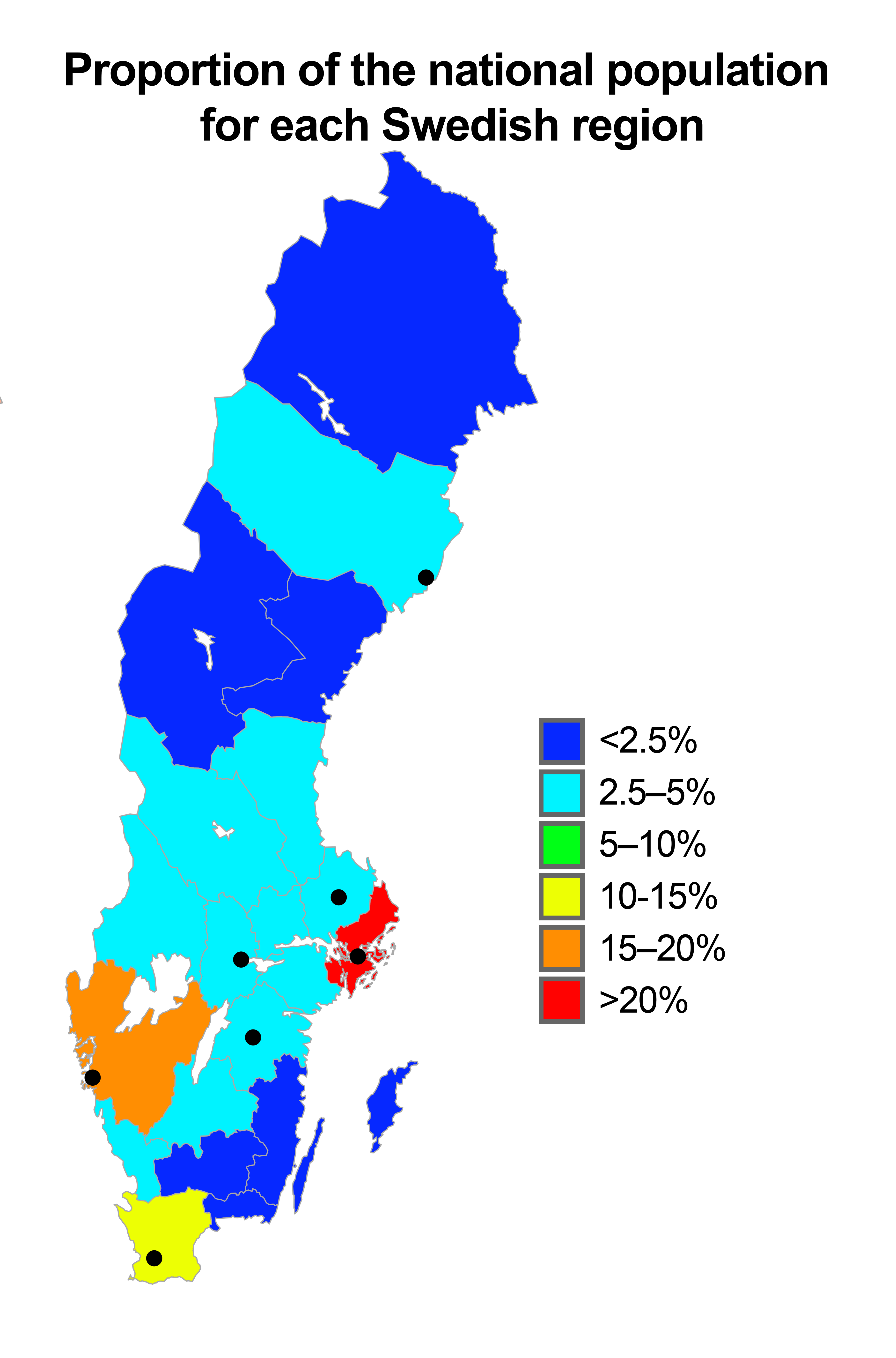

Supplement: sj-tif-1-eso-10.1177_23969873251347098 – Supplemental material for Large regional variation in endovascular thrombectomy rates for acute ischemic stroke in Sweden [file sj-tif-1-eso-10.1177_23969873251347098.tif]
